# Editorial: Education in public health: 2022

**DOI:** 10.3389/fpubh.2024.1385350

**Published:** 2024-02-28

**Authors:** Stefano Orlando, Enamul Kabir, Andrew Harver, Jie Hu

**Affiliations:** ^1^Dipartimento di Biomedicina e Prevenzione, Università degli Studi di Roma Tor Vergata, Rome, Italy; ^2^School of Mathematics, Physics, and Computing, University of Southern Queensland, Toowoomba, QLD, Australia; ^3^University of North Carolina at Charlotte, Charlotte, NC, United States; ^4^College of Nursing, Ohio State University, Columbus, OH, United States

**Keywords:** training curricula, education, public health, health professionals, active learning, emergencies, disasters

Public health, by virtue of its multidisciplinary nature, presents unique challenges in the education of students, researchers, and health professionals. The requisite skills must be drawn from a diverse array of fields, taking into account the complexity of factors that influence health and the effectiveness of interventions on populations (1). This comprehensive approach necessitates an educational framework that not only imparts knowledge across various disciplines but also fosters an understanding of their interplay in addressing public health issues. Such an education system is pivotal in equipping future public health leaders with the ability to devise and implement effective strategies that are responsive to the multifaceted determinants of health.

Another crucial aspect to consider in the education of those working in the field of public health is the mitigation of health disparities and the role of diversity and inclusion, particularly for minorities or discriminated populations. To address these issues effectively in both research and practice, a range of diverse competencies and soft skills is essential. This involves not only a deep understanding of the social determinants of health but also the ability to engage with communities in a manner that respects and values their unique perspectives and experiences. Cultivating such competencies enables public health professionals to develop and implement interventions that are culturally sensitive and equitable, thereby contributing to the broader goal of reducing health inequities and enhancing the wellbeing of all segments of the population.

In recent times, the COVID-19 pandemic has highlighted gaps in education and brought to the fore new challenges that have emerged. This unprecedented health crisis has underscored the necessity for public health curricula to be agile, adaptable, and responsive to emerging global health threats. The pandemic has not only exposed vulnerabilities in public health systems but has also called for a reassessment of how we prepare health professionals to navigate complex and rapidly evolving situations (2–5). The integration of pandemic preparedness, digital health technologies, and crisis management into public health education has become imperative. Such an approach ensures that future public health professionals are better equipped with the knowledge and skills needed to effectively respond to and manage health emergencies, thereby safeguarding the health and wellbeing of populations worldwide.

The Research Topic “*Education in public health: 2022*” has received a high number of papers, indicating significant interest in this theme. It is noteworthy that many submissions were not included in the Research Topic because they addressed health education more broadly, rather than focusing exclusively on individuals within the field of Public Health, and were therefore considered out of scope. However, this still underscores the complexity and relevance of education within the broader context of public health. The distinction between general health education and specialized education for public health professionals is crucial, yet the overwhelming response to this call for papers highlights the interconnectedness of these domains. It reflects a growing recognition of the need for comprehensive educational strategies that cater not only to public health professionals but also to the wider community, to foster a more informed and health-literate society capable of contributing to public health goals.

The Research Topic includes 17 articles, comprising nine Original Research Papers, three papers on Curriculum, Instruction, and Pedagogy, three Perspective papers, one Systematic Reviews, and one Community Case Study. The majority of the papers (13) are focused on student education, covering both the enhancement and evaluation of academic curricula and the extracurricular activities offered to students. In contrast, four papers are dedicated to the ongoing education of professionals already engaged in the workforce. This distribution underscores a predominant emphasis on shaping the foundational educational experiences of future public health professionals, while also acknowledging the importance of continuous learning and development for those actively contributing to the field. The diversity of article types reflects a comprehensive approach to exploring educational strategies, from theoretical frameworks and pedagogical innovations to empirical research and real-world applications, thereby providing a multifaceted perspective on education in public health.

An important theme is the development of what could be termed cross-disciplinary competencies or soft skills. Koh et al. discuss the integration of spirituality into public health leadership education at the Harvard Chan School. This article highlights the importance of spirituality in fostering resilience, meaning, and purpose among public health leaders, particularly in response to challenges encountered during the COVID-19 pandemic. It advocates for incorporating spiritual themes into public health leadership curricula to deepen understanding of personal motivations and enhance the ability to navigate complex public health issues. Landfried et al. examines the Master of Public Health (MPH) Capstone program at the University of North Carolina, which emphasizes community-led group projects. Utilizing a critical service-learning framework, the study assesses the program's effectiveness in benefiting students and community partners, highlighting the value of integrating service-learning into public health education. Horigian et al. research on the Learning Collaboratory at the University of Miami focuses on boosting public health students' skills through practical, community-oriented projects. The evaluation reveals enhancements in critical thinking, communication, problem-solving, collaboration, and leadership, pinpointing the strengths of a structured, collaborative approach while suggesting improvements. Dopelt et al. investigates the impact of simulation training on public health leadership skills development, employing a mixed-methods approach to evaluate how simulation scenarios enhance leadership, communication, and decision-making capabilities. The results affirm the effectiveness of simulation as a key educational tool in public health. Kedia et al. analysis of the U.S. job market for entry-level public health roles, based on a website job postings, and identifies the most sought-after skills and competencies, offering valuable insights for aligning public health education with employer expectations and workforce needs. The study of Hayes et al. presents a pedagogical framework and learning environment for the course, emphasizing public health finance and management. The population targeted includes doctoral-level students in public health. The main findings highlight the need for specialized training in public health finance and management for future leaders, addressing the gap in current academic offerings.

The theme of minority inclusion and racial issues is extensively explored in several papers. Sullivan et al. perspectivearticle discusses the “*Framing the Future 2030*” (FTF 2030) initiative by the Association for Schools and Programs of Public Health, which seeks to create a resilient educational system in public health. It emphasizes the initiative's focus on inclusive excellence through an anti-racism perspective, transformative teaching and learning methods, and broadening the field's scope. Valentim et al. evaluates a large-scale educational program's impact on prison health in Brazil, particularly its effects on the professional practices of healthcare workers in prisons. The study, through questionnaires, reveals notable improvements in professional practices and health services within prison settings, underscoring the efficacy of extensive, technology-enabled education in these contexts. Tagorda Kama leads two papers; the first examines the application of Critical Race Theory (CRT) in framing discussions around race and racism, validating experiential knowledge, and employing a social justice framework to tackle health inequities. This approach is designed to ready students for leadership roles in public health, emphasizing CRT's crucial role in addressing systemic racism and health disparities within public health education. The second paper outlines a program that engages high school students in public health education, especially those from diverse backgrounds, through various activities, coursework, and community projects. This study evaluates the program's success in sparking interest and developing public health skills among participants. Dutta and Keith investigate storytelling as a teaching method in a global health course at a Native American-Serving Non-tribal Institution, focusing on the transition from traditional to storytelling methods during the COVID-19 pandemic.

The COVID-19 pandemic has brought the issue of emergency preparedness to the forefront as a critical concern for both students and professionals in the field. Alsoukhni et al. evaluates the Public Health Empowerment Program (PHEP), focusing on its effectiveness in enhancing the skills of the public health workforce in the Eastern Mediterranean Region. The evaluation, based on Kirkpatrick's model and involving surveys of PHEP graduates and technical advisers, highlights the program's success in bolstering graduates' involvement in field epidemiology activities, especially during the COVID-19 pandemic. Fang's et al. systematic review assesses the current knowledge and skills of Chinese medical college students in managing public health emergencies and their training requirements. The findings reveal a general lack of capability among these students to handle public health crises, despite acknowledging the importance of such skills and expressing eagerness to learn. Kaim et al. investigates the effectiveness of the TEAMS 3.0 training package designed for Emergency Medical Teams (EMTs). Utilizing a pre-post study design to evaluate self-efficacy, teamwork, and training quality among participants from diverse countries, the study reports significant improvements in self-efficacy and teamwork following the training. This research underscores the importance of specialized training programs in public health education, particularly in improving the preparedness and performance of EMTs in disaster responses. Niu et al. investigates the risk perception of COVID-19 among Chinese university students using latent profile analysis and network analysis. The study reveals two main risk perception classes among students and examines the change in their risk perception over time. The findings suggest a decline in COVID-19 risk perception, emphasizing the role of cultural influence and effective government management.

One article is about evaluation of curricula's quality: Artyukhov et al. proposes a model for the external evaluation of medical education programs' quality, integrating various indicators such as international rankings, stakeholder input, and independent agency assessments. The study focuses on assessing the quality of medical education in relation to achieving sustainable development goals. It emphasizes the importance of both internal and external quality assurance in medical education and proposes a financial model for assessing educational program quality.

Wang et al. article aims to pinpoint the challenges encountered by public health professionals in their professional development. The study employs a methodological blend of association tests and logistic regression analyses, utilizing both traditional and data screening techniques to verify the validity of the data. This rigorous approach ensures a comprehensive understanding of the obstacles that public health professional face, contributing valuable insights to the field of public health education and workforce development.

## Author contributions

SO: Supervision, Writing—original draft, Writing—review & editing. EK: Writing—review & editing. AH: Writing—review & editing. JH: Writing—review & editing.
